# En-Face Optical Coherence Tomography Is Useful for Assessing Striated Lesions in Angioid Streaks: A Case Report

**DOI:** 10.7759/cureus.45983

**Published:** 2023-09-26

**Authors:** Takashi Takeuchi, Hiromasa Hirai, Nahoko Ogata, Tetsuo Ueda

**Affiliations:** 1 Ophthalmology, Nara Medical University, Kashihara, JPN

**Keywords:** anti-vegf vitreous injection, c-scan optical coherence tomography, en-face optical coherence tomography, choroidal neovascularization, angioid streaks

## Abstract

Angioid streaks are mainly characterized by radially striated lesions around the optical disc and result in severe vision loss when choroidal neovascularization (CNV) develops at the macula. The prediction of visual prognosis in cases with angioid streaks remains an unsolved problem. In this study, we report the usefulness of en-face optical coherence tomography (OCT) to assess the bilateral striated lesions in angioid streaks.

A 59-year-old female who was previously diagnosed with angioid streaks complained of decreased visual acuity in her left eye. However, on en-face OCT, the striated lesions in the right eye with better vision were shown as thicker continuous lesions than those in the left eye. Twenty-four months after the initial visit, her right visual acuity was worse than her left. En-face OCT showed fine-striated lesions extending from those thicker lesions to the macular area in the right eye. The thicker striated lesions observed at the initial visit may be a risk factor for future CNV development and vision loss. The evaluation of lesion size using en-face OCT may be useful for predicting the visual prognosis in angioid streaks.

## Introduction

Angioid streaks develop bilaterally in middle-aged patients and are characterized by striated lesions resulting from Bruch's membrane fissures [1]. These striated lesions are known to extend radially from the optical disc. Although angioid streaks are usually asymptomatic, choroidal neovascularization (CNV) from the striated lesions may result in severe visual loss. Anti-vascular endothelial growth factor (VEGF) vitreous injections are widely used for the treatment of CNV secondary to angioid streaks [1,2]. However, predicting the visual prognosis in cases with angioid streaks remains an unsolved problem. The striated lesions have been examined using fundus photography, fluorescein angiography, and B-scan optical coherence tomography (OCT) focusing on the macula [1]. These conventional methods make it difficult to evaluate the three-dimensional (3D) structure of striated lesions. En-face OCT has recently become a popular method for observing continuous lesions by extracting desired layers from the frontal view [3]. There have been few reports of using en-face OCT for the evaluation of angioid streaks. In this study, we report a case in which en-face OCT was useful for assessing the structure of continuous striated lesions in angioid streaks.

## Case presentation

**Figure 1:**
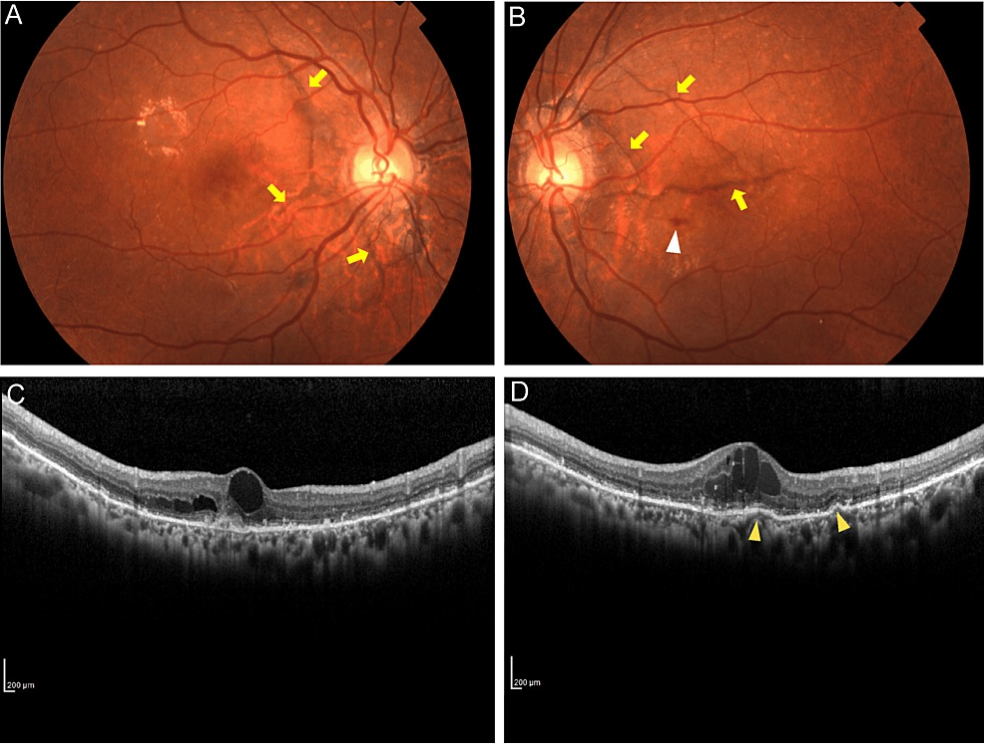
Fundus photographs at the initial visit, A (right eye) and B (left eye), and B-Scan OCT of the fovea at the horizontal section, C (right eye) and D (left eye) A: Right eye. Angioid streaks (striae radiating from the optic disc area to the peripapillary area) are shown (yellow arrows). B: Left eye. Angioid streaks (striae radiating from the optic disc area to the peripapillary area) are shown (yellow arrows). Retinal hemorrhage at the macula (white arrowhead) is observed. C: Right eye. The macular edema and intraretinal hyperreflective foci are seen. D: Left eye. The macular edema and the elevations of the retinal pigment epithelium layer (yellow arrowheads) are detected. The intraretinal hyperreflective foci are more frequent in the left eye. OCT: optical coherence tomography

A 59-year-old female complained of progressive left eye vision loss one month ago and visited our hospital. She did not have general symptoms. At the initial visit, her decimal best-corrected visual acuity (BCVA) was 1.0 with +0.75-1.00 × 90 in the right eye and 0.7 with +0.75 in the left eye. The intraocular pressure was 16 millimeters of mercury in the right eye and 15 millimeters of mercury in the left eye. The refractive error (spherical equivalent) determined by an auto refractometer (ARK1a®, NIDEK, Aichi, Japan) was +0.25 diopters (D) in the right eye and +0.375 D in the left eye. Her eyelids and conjunctivas were normal. Slit-lamp examinations showed that the corneas were clear, the anterior chambers were of normal depth, and the lenses were transparent in both eyes. Fundus examination revealed angioid streaks in both eyes and macular hemorrhage in the left. On fundus photographs (TRC-50DX®, TOPCON, Tokyo, Japan), striated lesions in both eyes seemed to be comparable (Figures 1A-1B). B-Scan OCT (Spectralis®, Heidelberg Engineering, Heidelberg, Germany) at the fovea displayed macular edema and hyperreflective foci in both eyes and those were more abundant in the left (Figures 1C-1D).

**Figure 2:**
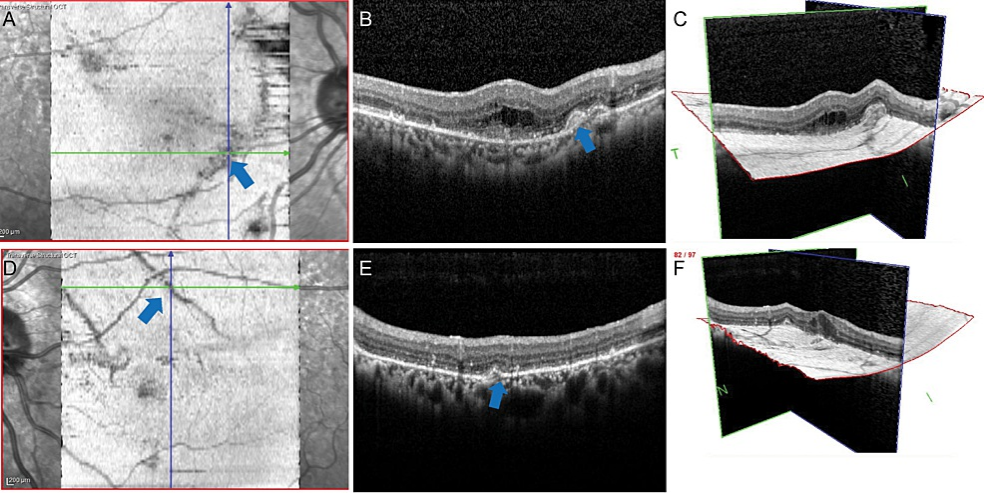
En-face OCT images at the initial visit. In the right eye, A (transverse scan), B (horizontal scan), and C (3D view). In the left eye, D (transverse scan), E (horizontal scan), and F (3D view) A: The striated lesions in the right eye are clearly shown in the transverse scan. B: Horizontal scan of the striated lesion in the right eye. The lesion is shown to be elevated (blue arrow, same location as A). C: Total 3D view of the striated lesion in the right eye. The striated lesion is shown to be continuous (same location as A and B). D: The striated lesions in the left eye are also clearly shown in the transverse scan. E: Horizontal scan of the striated lesion in the left eye. The lesion is smaller than that in the right eye (blue arrow, same location as D). F: Total 3D view of the striated lesion in the left eye. The striated lesion is continuous but smaller than in the right eye (same position as D and E). OCT: optical coherence tomography

En-face OCT at the Bruch membrane level clearly showed striated lesions within the arcade in both eyes (Figures 2A-2D). The height of the striated lesion was higher in the right eye than in the left (Figures 2B-2E). Furthermore, the 3D mode revealed that the striated lesions were thicker "continuous" lesions in the right eye (Figures 2C-2F).

**Figure 3:**
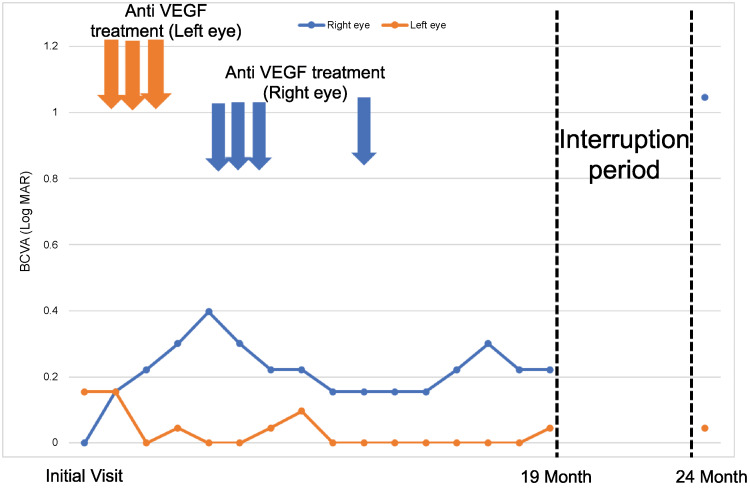
Time course of the treatment in both eyes Vertical axis of the line graph: BCVA (Log MAR). Horizontal axis of the line graph: time course. Blue line: visual acuity trend in the right eye. Orange line: visual acuity trend in the left eye. After five months of interruption, the right visual acuity was significantly worsened. In contrast, the left visual acuity was maintained. BCVA: best-corrected visual acuity, VEGF: vascular endothelial growth factor

We performed three times of anti-VEGF vitreous injections, aflibercept (Eylea, Bayer HealthCare Pharmaceuticals, Berlin, Germany), in the left eye (Figure 3). The drugs were administered 4.0 mm posterior to the corneal limbus at a dose of 2.0 mg/0.05 mL. Four months after the initial visit, the macular edema was resolved in the left eye, and her left BCVA was improved to 0.9. However, the macular edema increased in the right eye, and her right BCVA decreased to 0.5. We performed four times of anti-VEGF vitreous injections in the right eye. Nineteen months after the initial visit, her right BCVA was 0.6, and her left BCVA was 0.9. Her right eye vision worsened from the initial visit. On the other hand, her left eye vision had been maintained after the initial three vitreous injections.

**Figure 4:**
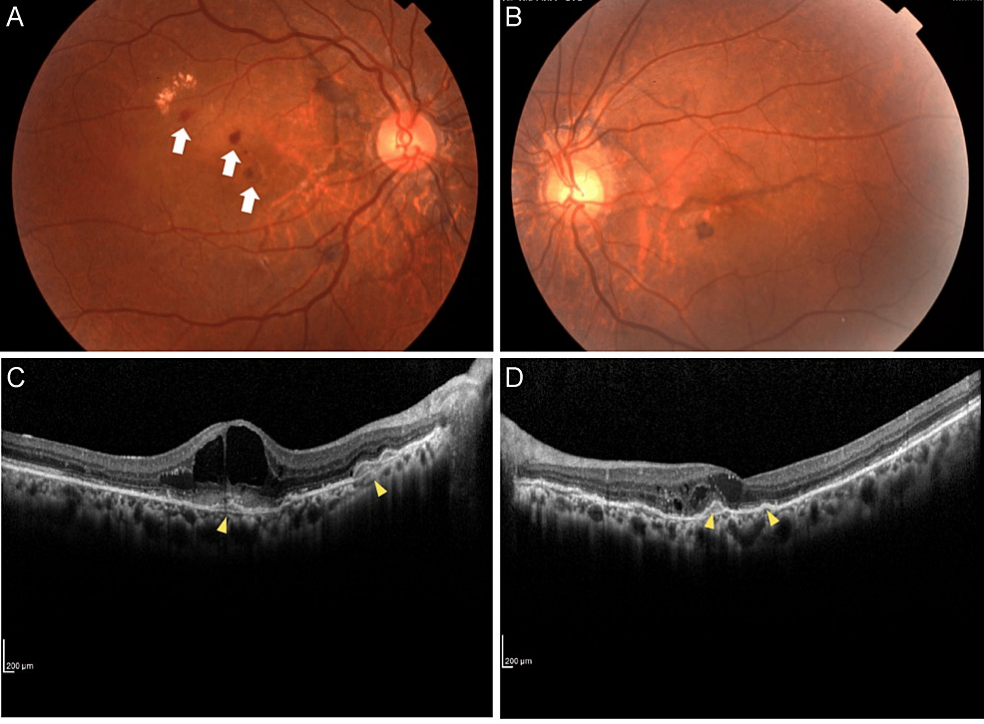
Fundus photographs 24 months after the initial visit, A (right eye) and B (left eye), and B scan OCT of the fovea at the horizontal scan, C (right eye) and D (left eye) A: Right eye. Several retinal hemorrhages are seen (white arrows). B: Left eye. There is no apparent hemorrhage. C: Right eye. The edema is worsening and there are elevations of the retinal pigment epithelium layer (yellow arrowheads) that were not present two years ago. D: Left eye. A slight edema is recognized, but there is no remarkable change in the elevation of the retinal pigment epithelium layer compared to two years ago. OCT: optical coherence tomography

She developed a cerebral infarction at that time, and her follow-up was interrupted. Twenty-four months after the initial visit, she visited our hospital again. Her right BCVA had decreased to 0.09. On fundus examination, several macular hemorrhages were revealed on the right (Figures 4A-4B). On B-Scan OCT at the fovea, the macular edema was worse, and new elevations of the retinal pigment epithelium layer were seen in the right eye (Figures 4C-4D).

**Figure 5:**
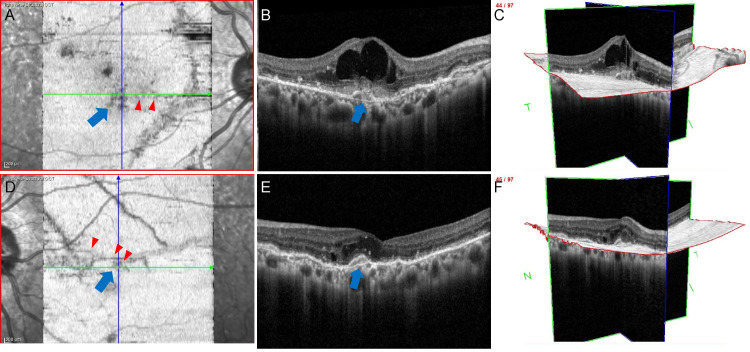
En-face OCT images 24 months after the initial visit. In the right eye, A (transverse scan), B (horizontal scan), and C (3D view). In the left eye, D (transverse scan), E (horizontal scan), and F (3D view) A: Fine-striated lesions are seen extending from the thick striae to the macula (red arrowheads). B: Horizontal scan of the macula area in the right eye. The lesion is shown to be elevated (blue arrow, same location as A). C: Total 3D view of the striated lesion in the right eye. The striated lesion is shown to be continuous (same location as A and B). D: Fine-striated lesions also extend to the macula in the left eye (red arrowheads). E: Horizontal scan of the striated lesion in the left eye. The lesion is smaller than that in the right eye (blue arrow, same location as D). F: Total 3D view of the striated lesion in the left eye. The striated lesion is continuous but smaller than in the right eye (same position as D and E). OCT: optical coherence tomography

En-face OCT at the Bruch membrane level showed fine-striated lesions extending from the previously observed thick lesions to the macular area on the right (Figure 5). The elevations of the retinal pigment epithelium layer observed by B-scan OCT were confirmed to be striations. In the left eye, a small stria was also observed around the macula. Fortunately, her left BCVA remained at 0.9 and the macular edema was slight.

## Discussion

Angioid streaks often indicate connective tissue disease, associated with cutaneous pseudoxanthoma elasticum [4]. However, some patients present with ocular findings only, as in our case. The radial striated lesions of angioid streaks reflect calcification and disrupted elastic fibers within Bruch’s membrane [1]. CNV, which occurs in the late stages of angioid streaks, is derived from those radially striated lesions. Several reports have described the association between hyperreflective foci and the activity of angioid streaks in B-scan OCT [5,6]. In the present case, intraretinal hyperreflective foci were found to be more abundant in the left eye at the initial examination. Those intraretinal hyperreflective foci are thought to be associated with the accumulation of intraretinal fluid and, thus, reflect present CNV. However, the imaging features of angioid streaks reflecting the long-term prognosis remain unknown. Anti-VEGF vitreous injections are currently widely used to treat CNV in angioid streaks. However, due to the fragility of Bruch's membrane, CNV recurs and additional injections are often required. The destruction of Bruch’s membrane and invasion of choroidal fibrous tissue in the striae on OCT have been previously reported [7]. According to this study, the invasion occurs in more advanced angioid streak cases. Thus, the degree of choroidal tissue invasion reflects the extent of Bruch’s membrane damage at that site. Although Risseeuw et al. reported the classification of angioid streak severity according to the length of the striated lesions [8], there have been no previous reports classifying them according to the depth of the lesions.

En-face OCT is an imaging technique for viewing lesion extension and depth. It has been used for the diagnosis of age-related macular degeneration [3], but there have been few reports on its use for the diagnosis of angioid streaks. Hanhart et al. have recently used en-face OCT to identify fine-striated lesions that seem to be the cause of CNV [9,10]. In our case, new small lesions could be identified by en-face OCT after two years of follow-up. Furthermore, we clearly demonstrated that the striated lesions at the initial visit were thicker in the right eye than in the left eye by using 3D imaging. Fundus photographs could not show differences between the two eyes. Interestingly, the visual prognosis was worse in the right eye, which had thicker lesions. This may suggest that new striations and CNVs may develop from the elevated lesions over time.

## Conclusions

In summary, we report the application of en-face OCT to the evaluation of striated lesions in a patient with angioid streaks. En-face OCT can visualize the thickness of striated lesions at any desired location within the arcade, which is helpful for observing the pathogenesis of angioid streaks. A thick striated lesion near the arcade may be a risk for future vision loss due to the development of CNV. Further studies are needed with a larger number of cases.
